# A closer look to the timing of orchidopexy in undescended testes and adherence to the AWMF-guideline

**DOI:** 10.1007/s00383-024-05659-3

**Published:** 2024-02-29

**Authors:** C. Von Cube, A. Schmidt, M. Krönninger, G. Hrivatakis, W. Astfalk, J. Fuchs, V. Ellerkamp

**Affiliations:** 1 Department of Pediatric Surgery and Pediatric Urology, Eberhard Karls University, Tubingen, Germany; 2 Outpatient Clinic for Pediatric and Adolescent Surgery, Stuttgart, Germany; 3 Outpatient Clinic for Surgery, Reutlingen, Germany; 4 Department of Pediatric Surgery, Municipal Hospital Karlsruhe, Karlsruhe, Germany

**Keywords:** Cryptorchidism, Guidelines, Orchidopexy, Pediatric surgery, Undescended testis

## Abstract

**Background:**

To lower the risk of testicular malignancies and subfertility, international guidelines recommend orchidopexy for undescended testis (UDT) before the age of 12–18 months. Previous studies reported low rates of 5–15% of timely surgery. Most of these studies are based on DRG and OPS code-based data from healthcare system institutions that do not distinguish between congenital and acquired UDT.

**Methods:**

In a retrospective study data of all boys who underwent orchidopexy in a university hospital and two outpatient surgical departments from 2009 to 2022 were analyzed. The data differentiates congenital from acquired UDT.

**Results:**

Out of 2694 patients, 1843 (68.4%) had congenital and 851 (31.6%) had acquired UDT. In 24.9% of congenital cases surgery was performed before the age of 12 months. The median age at surgery for congenital UDT was 16 months (range 7–202). Over the years there was an increased rate of boys operated on before the age of 2 (40% in 2009, 60% in 2022). The median age fluctuated over the years between 21 and 11 months without a trend to younger ages.. The covid pandemic did not lead to an increase of the median age at surgery. The median time between referral and surgery was 46 days (range 1–1836). Reasons for surgery after 12 months of age were a delayed referral to pediatric surgeries (51.2%), followed by relevant comorbidities (28.2%).

**Conclusion:**

Compared to recent literature, out data show that a closer look at details enables a more realistic approach. Still, there is no trend towards the recommended age for surgical treatment observable, but the rate of timely operated boys with congenital UDT is significantly higher than stated in literature.

**Supplementary Information:**

The online version contains supplementary material available at 10.1007/s00383-024-05659-3.

## Introduction

Congenital undescended testis (UDT) is the most common urogenital anomaly of newborn boys with a prevalence ranging from 1% to 8.4% [1]. If left untreated, UDT may cause infertility in adulthood [2, 3]. Additionally, some studies suggest an increased predisposition of testicular cancer, which can be significantly reduced by prepubertal orchidopexy [3, 4]. Before or instead of surgery, preoperative hormonal treatment with human chorionic gonadotropin (hCG) and gonadotropin-releasing hormone (GnRH) may be considered [5]. However, it is important to note that the effectiveness of this treatment is not as well-established as that of surgical treatment and it is rarely recommended in practice. Additionally, the European Association of Urology (EAU) does not recommend this treatment as a means of preservingfuture fertility [6].

In 1996, the American Academy of Pediatrics (AAP) recommended orchidopexy at around the 12 months of age [7]. This recommendation was confirmed by the Nordic Consensus in 2007 and subsequently followed by the German AWMF-guideline in 2009, as well as the European Association of Urology (EAU) [8]. However, according to EAU guidelines and the cryptorchidism guidelines of the American Urological Association (AUA), the latest accepted time for orchidopexy is the age of 18 months [6]. Several studies have shown that there is significant room for improvement in terms of timely operation according to the guidelines [8–12].

In Germany, health examinations for children and adolescents are recommended as benefits of the statutory health insurance. In the state of Baden-Württemberg, where this study was conducted, the Child Protection Act (Kinderschutzgesetz Baden-Württemberg) prescribes obligatory participation in early preventive examinations for children (U1–U9, J1). This obligation aims to increase attendance of early preventive examinations for children and adolescents. The examination of the testicular position is a crucial component of the general physical examination from U2 onwards. The aim of our ongoing data evaluation was to analyze whether there has finally been an adequate improvement in the implementation of the above-mentioned German guidelines, to evaluate possible reasons for delayed surgery and to compare our findings with similar studies.

## Materials and methods

A retrospective analysis of the data of boys with UDT was performed including data from 2009 to 2022 of a university hospital (University Hospital Tuebingen, Department of Pediatric Surgery and Pediatric Urology) and from 2009 to 2020 of two outpatient clinics (Outpatient Clinic for Pediatric and Adolescent Surgery, Stuttgart, Dr. med. Hrivatakis; Outpatient Clinic for Surgery, Reutlingen, Dr. med. Astfalk). The patient collective was selected on the basis of the diagnosis code of the International Statistical Classification of Diseases and Related Health Problems, 10th revision, with the German modification ICD-10-GM and the German operation and procedure code (OPS). Patients with the following codes were recorded: Q53.0, Q53.1, Q53.2 and Q53.9 as well as 5–622, 5–623, 5–624, 5–625, 5–626. The data was analyzed by two researchers. Relapse orchidopexy, or simultaneous orchidopexy as part of a required herniotomy within the first 6 months of life were excluded with the aim to avoid bias of median age at surgery. The data differentiates congenital from acquired UDT by anamnestic evaluation, as the testicular position is examined by pediatricians as part of the in Baden-Württemberg legally required early preventive examinations for children. Furthermore, the affected side, and preoperative hormonal treatment (YES, NO) were recorded. Based on the German AWMF recommendations concerning the recommended age of orchidopexy, patients were divided into the following age groups: Surgery at < 12 months, 12–24 months, or > 24 months.

More detailed data concerning exact ages at surgery and referral were available for the years 2009–2022 (university hospital) and 2016–2020 (outpatient institutions).

Data sheets from the university hospital were also analyzed concerning the following details: prematurity (YES, NO), hydatid of Morgagni (YES, NO, NOT MENTIONED); anatomy of the testis (NORMAL, TESTIS-EPIDIDYMIS-DISSOCIATION, TESTICULAR RUDIMENT), intraoperative location of the testis (INGUINAL LOW, INGUINAL MIDDLE, INGUINAL HIGH, ABDOMINAL, EPIFASCIAL, SCROTAL).

The study was approved by the state medical association of Baden-Wuerttemberg as well as by the Ethic committee of the University of Tuebingen, Germany (No 064/2020BO2).

For descriptive statistical analyses SPSS Statistics 26 (IBM) was used. The evaluated data were described with absolute and relative frequencies, as well as examined in cross tables. An exploratory data analysis of the recorded characteristics and concomitant symptoms was additionally performed. Normality of data was tested with Shapiro–Wilk and Kolmogorov–Smirnov test. Non-parametric data were expressed as median with range. Parametric data were expressed as mean with standard deviation. Significance of two nonparametric groups, the Mann–Whitney *U* test was applied at the 5% significance level. Univariate analysis of the data and possible correlations between nominal and ordinal characteristics were followed up in cross-tabulations and tested using two-sided Fisher's exact test and the chi-square test at a 5% significance level.

## Results

Data sets of 2694 boys (3314 affected testes) with UDT and orchidopexy were available. Out of these, 1583 boys (58.76%) were treated in outpatient institutions (1005 boys in the outpatient Clinic in Reutlingen, 578 boys in the outpatient clinic in Stuttgart), while 1111 (41.24%) were treated in the university hospital. In 1843 boys, UDT was congenital (right: 42.82%, left: 31.41%, bilateral 25.77%), and 851 boys had acquired UDT (right: 52.06%, left: 30.90%, bilateral 16.04%) with an anamnestic history of initial scrotal position during the first preventive medical checkups for children. The rate of congenital UDT was higher in hospitals compared to outpatient services (74.8% vs. 63.93%, *p* < 0.001). The numbers of orchidopexies varied over the years, only the cases of acquired UDT in the hospital remained stable: in the hospital, the mean number per year of boys with congenital UDT was 59.21 (15.3), the mean number of acquired UDT was 20.14 (4.4). In the outpatient services, the mean number of boys with congenital UDT was 84.3 (16.61), the mean number of acquired UDT was 51.91 (20.84). The lowest count of all UDT was in 2016 due to a major decline of UDT in the outpatient services. The highest count of all UDT was in 2018, as the result of an increase both in the hospital and in the outpatient practice. Lowest case numbers were accounted in the hospital from 2020 to 2022 (Supplementary file 1). Preoperative hormonal treatment was registered in 264 boys with congenital UDT (13.62%), and in 28 boys with acquired UDT (3.26%).

**Fig. 1:**
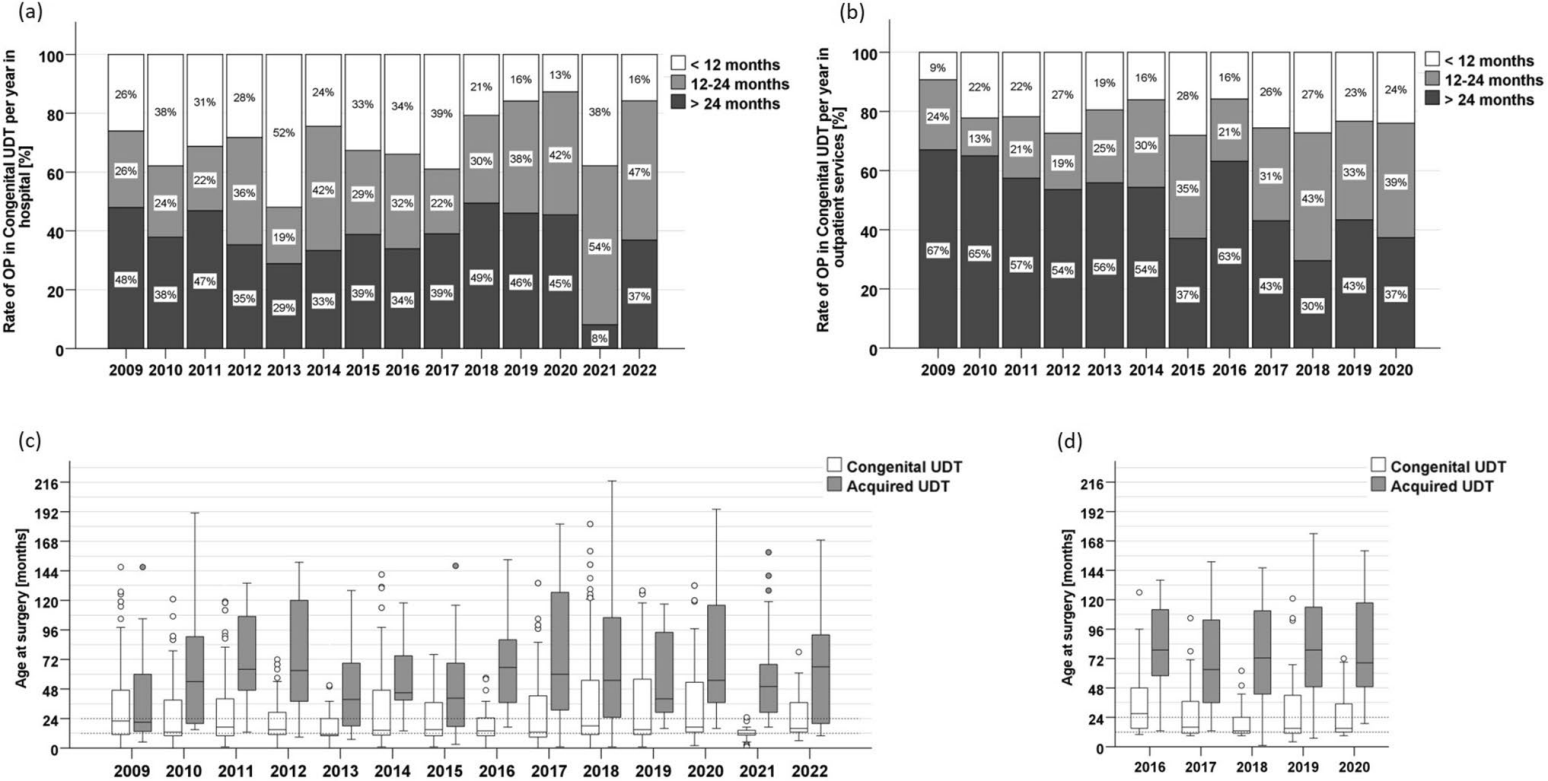
Age groups and median ages at orchidopexy. Rates of age groups per year of orchidopexies in congenital UDT per year of the hospital (**a**), and of the outpatient services (**b**). Median ages of congenital UDT (white boxplots) and acquired UDT (grey boxplots) of the university hospital (**c**) and the outpatient services (**d**)

The rate of boys with congenital UDT and orchidopexy before 12 months of age was 24.93%, 29.33% were operated on between 12 to 24 months and 45.74% after their second birthday. Over the years, the rate of boys with congenital UDT and orchidopexy after the age of 24 months decreased from 58.82% to 36.84%, consecutively, the rates of OP before the age of 24 months increased from around 40–63% in the hospital as well in the outpatient services. However, there was no steady increase in operations on children under one year old: in 2009 the rate started with 16.5%, rose to rates around 25 to 30% during the years 2010–2018 and the decreased to 19% in 2020 and 16% in 2022. In the hospital, the rates of boys with congenital UDT and surgery before the age of 12 months were slightly higher than in the outpatient services (Fig. 1). During the pandemic, less orchidopexies were counted, but no increase of higher age groups.

Focusing on exact ages at surgery, data were available for all patients treated in the hospital. For the outpatient services, these data were only available for the cohorts of 2016 to 2020 (Supplemental Table 1, Fig. 1). The median ages at surgery for congenital UDT ranged between 12 and 25 months and were in the lower range during the pandemic (Fig. 1).

For congenital UDT, the median time between pediatric referral to surgery was significantly higher in the outpatient service compared to the hospital with (*p* < 0.001); there was no significance comparing this period between congenital and acquired UDT (Supplementary file 2). In the hospital during the pre-pandemic period (2009–2019), the median time between referral and surgery was 39 days (1–1836) and increased significantly to 77.5 days (1–1044) during the pandemic period (2020–2022) (*p* < 0.001).

Anatomical and clinical details were available for all 1111 hospital patients, including 1363 testes. In the cohort with congenital UDT treated in hospital, 35% boys were operated on without delay (median age of 10 months (7–12)) after timely admission (median age of 8 months (0–12)). 65% cases were operated on after their first birthday. Reasons for delay in this subgroup were the following: in 51.4%, the boys were only transferred after the age of 12 months (median: 36 months (12–196), resulting in a median age at surgery of 37 months (13–202). In 28.2% relevant comorbidities led to a delay of surgery with a median age of 35 months (13–200). Relevant comorbidities were severe syndromes, congenital metabolic diseases or neonatal complications in 87 cases, neonatal surgery in 35 cases, hypospadias in 20, and oncological diseases in 6 cases. Further reasons for delay were unknown in 12.1%, uncertainty in differentiation between retractile and congenital UDT in 4,8%, parental doubts in 1.1%, immigration in 0.7%, pandemic reduced capacity in 0.7%, hormonal therapy in 0.6%, and acute contraindications 0.4%.

Prematurity was found in 133 boys, boys with congenital UDT were significantly more often affected (61.7%, *p* < 0.005). A hydatid of Morgagni (appendix testis) was found in 52.3% of congenital UDT and in 55.3% of acquired UDT. Similar equality was found in the anatomical findings of the testis: in cases with congenital UDT 60.6% had a normal anatomy, a dissociation was described in 36.8%, 2.6% had only a testicular rudiment; in acquired UDT, 74.0% had a normal anatomy, 25.7% had a testicular dissociation and 0.3% had a rudiment. A persistent processus vaginalis was described in significantly higher rates in congenital UDT (44.02%) than in acquired UDT (32.82%, *p* < 0.001). In 23.26% of the acquired UDT, the boys had inguinal hernia surgery of the affected side in their medical history, while this was only in 2.03% of congenital UDT the case (*p* < 0.001).

Information concerning the intraoperative position of the testis was available for 1129 testes (Supplementary file 3). The majority all UDT (31,1%) was located epifascial or inguinal middle (29.2%). In 110 testes, a two-stage Fowler-Stevens operation was performed. In 48 cases, a testicular remnant was resected and no orchidopexy could be performed.

## Discussion

Since scientific recommendations regarding the timing of orchidopexy in UDT were released, numerous studies have dealt with their implementation, starting in the early 1960s [4, 7]. Prior to these recommendations only a small percentage of boys (5%) underwent surgery before their second birthday, with a high mean age.However, since the early 1990s, these rates have significantly improved, ranging from 17 to 64% [16, 18, 22]. The rate of orchidopexies before the completion of the first 12 months of life ranged from 3 to 24% [19, 21]. Few studies adhere to the AUA recommendation of reporting rates of orchidopexy before the 18th month of life. The reported rates range from 16% to even 87%. However, these studies exclude orchidopexies performed after the age of 5 years in an attempt to exclude acquired cases [24, 25]. Overall, no clear improvement trend over time was reported highlighting the discrepancy between the recommendations and reality. In 2009, a multicentric approach was initiated to collect data on UDT. Our study revealed low rates (18.7%) of timely orchidopexy before the age of 12 months [8]. Continuous data analyses of our study group over the years have shown no relevant changes in these rates [9]. In our recent update, the rate of timely surgery before the age of 12 months in congenital cases remains low at approximately 25%, but it is higher than in all other comparable studies. In contrast, in a recent German study of InEK and BNKD data (Institute for the Remuneration System in Hospitals, professional association of pediatric surgeons) only 15% of patients treated in hospitals and only 5% of those treated in outpatient services were younger than one year [20].

Classifying solely based on age groups alone, carries the risk of imprecision, and heterogeneous age groups make a comparison challenging [11, 13, 17, 20, 23, 24]. Therefore, a closer look at the exact ages of the individuals is necessary. However, most studies that investigate the exact ages at surgery only provide the mean age, without any information about previous testing for normal distribution (Table 1).This lack of information hampers the interpretation of the data [4, 14–16, 18, 21, 22, 26–28]. The mean ages reported ranged from 44 to 64 months [15, 27] with an upward aberration from periods before the recommendations of the pediatric urologic associations of > 100 months [4], and one downward aberration in a very small cohort of only 88 cases with 23 months [16]. For comparative purposes, we calculated the mean age of our overall cohort to be 34 months. The median age at orchidopexy of 16 months for congenital cases in our cohort was similar to the AUA recommendation and markedly better than the median ages reported in other studies, which ranged from 19 to 30 or even 60 months [10, 19, 25].

**Table 1:** Overview of studies concerning ages at orchidopexy

	Nation	Number of patients	Distinction cUDT vs. aUDT	Data resource	Analysed years	Orchidopexy			Mean age at surgery [months]	Median age at surgery [months]	
						< 12 months (%)	< 18 months (%)	< 24 months (%)		Congenital UDT	Acquired UDT
Pettersson A, 2007 [4]	S	16,983	No	NH-data	1964–1999			5	103.2		
Steckler ER, 1995 [22]	USA	329	No	Multicentre	1991—1993	17		44	51.6		
Upadhyay V, 2001 [26]	NZ	325	No	Single centre	1996–1998				52.2		
Lim KT, 2003 [15]	IRL	97	No	Single centre	1996–1999			29	67.2		
McCabe JE, 2008 [17]	GB	36,847	No	NH-data	1997–2005			24			
Bruijnen CJP, 2008 [13]	NZ	788	No	Multicentre	1997–2007			43			
Kokorowski PJ, 2010 [14]	USA	28,204	No	NH-data	1999–2008	18		43	52.8		
Springer A, 2013 [21]	A	19,998	No	NH-data	1993–2009	3		23	62.4		
Chen YF, 2013 [27]	CN	547	No	NH-data	1997–2009				44.7		
Zöller G, 2005 [23]	D	127	Yes	Single centre	2002–2004			13			
Yiee JH, 2012 [24]	USA	1365	(Yes)^a^	NH-data	2002–2007		87%				
Bayne AP, 2011 [28]	USA	677	No	Single centre	2002–2009				50.4		
Moslemi MK, 2014 [18]	IR	252	No	Single centre	2005–2009			17	53.3		
Nah SA, 2014 [19]	SGP	513	Yes	Single centre	2007–2011	23		58		19.2	
Baskovic M, 2022 [25]	CR	198	Yes	Single centre	2019		16%			30	99
Marchetti F, 2012 [16]	I	88	No	Multicentre	2002–2004	13		64	22.8		
Williamson SH, 2022 [11]	USA	17,012	(Yes)^a^	NH-data	2000–2021		30–55%				
Williams K, 2017 [10]	USA	2482	No	NH-data	2012			36		48	
					2015			32		60	
Schmedding A, 2023 [20]	D	9731	No	NH-data	2005–2020	7–12		34–55			
Own data 2023	D	2694	Yes	Multicentre	2009–2022	25		54			
		1757		Multicentre	2009–2022				34.1	16	63

*cUDT* congenital UDT, *aUDT* acquired UDT, *NH-data* data of national health systems, insurances

E.g.; ^a^Exclusion of boys with orchidopexy > 5 years

In Germany, the first version of a guideline on the diagnosis and treatment of undescended testicles was published by the AWMF in 1999 with the recommendation of orchidopexy before the second birthday. Since then, it has been continuously updated in line with the latest scientific findings. The last two versions of the guideline, from 2009 and 2016, recommend treatment between the 6th and 12th month of life for primary undescended testicles [8, 29, 30]. In 2009, we started our evaluation to analyze the impact of the guideline recommendation on the clinical practice, but in fact the expected effect of significantly earlier orchidopexy could not be reproduced.

As a small aside, the risk of distorting the results by simply looking at age groups is already apparent in the focus on the pandemic years 2020 to 2022. In our cohort, the pandemic did result in less orchidopexies per year in 2020ß to 2022, a markedly longer time from referral to surgery, lower rates of orchidopexies in boys younger than 12 months, but not in higher median ages. The reduction of elective surgeries due to reduced surgical capacity during the pandemic and the hesitancy of some parents to schedule operations that may not have been perceived by them as urgent may be one explanation of the lower rates [31]. Furthermore, orchidopexies in children well beyond their first birthday may therefore have been considered less urgently in the allocation of surgical appointments than those in whom an orchidopexy could be performed in a timely manner. It may be anticipated that the number of orchidopexies will increase significantly in the following years, with the median age at surgery increasing at the same time. This will be investigated in the following studies.

The reason for the better results in our study regarding the age of orchidopexy, is due to the distinction made between congenital and acquired UDT. However, distinguishing between congenital and acquired UDT is discussed controversially. In the past, some authors believed that acquired UDT was actually an unrecognized congenital UDT [32]. In contrast, a prospective longitudinal population-based child cohort study of over 1000 boys showed that acquired UDT accounts for up to 58% of all cases of cryptorchidism. These findings are supported by several other studies [33]. In the context of this data, the collected data on acquired and congenital UDT of our current study is comprehensible. However, it is important to note thatthe anamnestic recording may be subject to some degree of error. For the early preventive examinations for children (U1–U9, J1), an obligation to participate is anchored in the Child Protection Act of Baden-Württemberg (§ 1 para. 1 sentence 1 KiSchG BW). From U2, which is typically conducted between the 3rd and 10th day of life, the genital and thus the testicular position is regularly examined as part of the thorough physical examination and deviations are noted accordingly. Acquired UDT results from inadequate longitudinal growth, restraining fibrous portions, spermatic cord or other factors such as iatrogenic after inguinal hernia surgery, that occurs at newborn age [33]. Studies on timing of orchidopexy are mostly based on DRG and OPS code-based data from health insurance companies or national healthcare system institutions, which do not differentiate between congenital and acquired UDT. Therefore, it is likely that the actual age of patients with congenital UDT is lower in these cohorts. We found no significant anatomical differences (e.g. hydatid or testis-epididymis-dissociation) between acquired and congenital UDT. A marked majority of cases of acquired UDT were located epifascial or in the low inguinal canal, while a slight majority of congenital cases were found in the middle and high inguinal canal or abdominal. The fact of higher rates of prematurity in boys with congenital UDT was not surprising.

However, to further reduce the median age of orchidopexy in congenital UDT,optimizing pediatric referral patterns is crucial. Our study, similar to other studies, identified a late referral as a primary reason for delayed surgery. High rates of late presentation have already been reported, indicating a need for modifications of the guidelines to address this issue [12, 25, 27, 34]. Second most likely comorbidities of boys led to delayed orchidopexy. The avoidance of multiple anesthesia for different operations certainly plays a relevant role and can only be handled differently to a limited extent. In addition, serious health restrictions can relativize the relevance of a timely orchidopexy in individual cases.

In summary, the herein reported cohort revealed a high rate of orchidopexies in congenital UDT below the age of 24 months and the highest rate of orchidopexies below the age of 12 months as well as the lowest median age compared to literature. This is certainly based on the exclusion of the acquired UDT, but there may also be a bias towards earlier orchidopexies due to ongoing years of data collection and evaluation in our centress. The fact that early preventive examinations in childhood are legally mandated in Baden-Württemberg may also contribute to a sbetter guideline consistency. As age at orchidopexy may be an indicator of the quality of the respective regional health service, significant regional variations are described in other studies [13].

The study is limited by its retrospective nature, which is a common limitation in studies on age at orchidopexy. However, the detailed data acquisition with discrimination between acquired and congenital UDT balances this limitation. by. In addition, the data is restricted to a small geographical area and a specific group of pediatric surgical/urological units in Germany, which limits the ability to compare with other units in different parts of the country.

## Supplementary Information

Below is the link to the electronic supplementary material.

Supplementary file1 (JPG 144 KB)

Supplementary file2 (DOCX 15 KB)

Supplementary file3 (DOCX 12 KB)

## Data Availability

The datasets generated during and/or analysed during the current study are available from the corresponding author on reasonable request.
