# Supplementary figures and images for: A closer look to the timing of orchidopexy in undescended testes and adherence to the AWMF-guideline
Source: Pediatr Surg Int. 2024 Feb 29;40(1):60. doi: 10.1007/s00383-024-05659-3 (PMC10904547; doi:10.1007/s00383-024-05659-3)

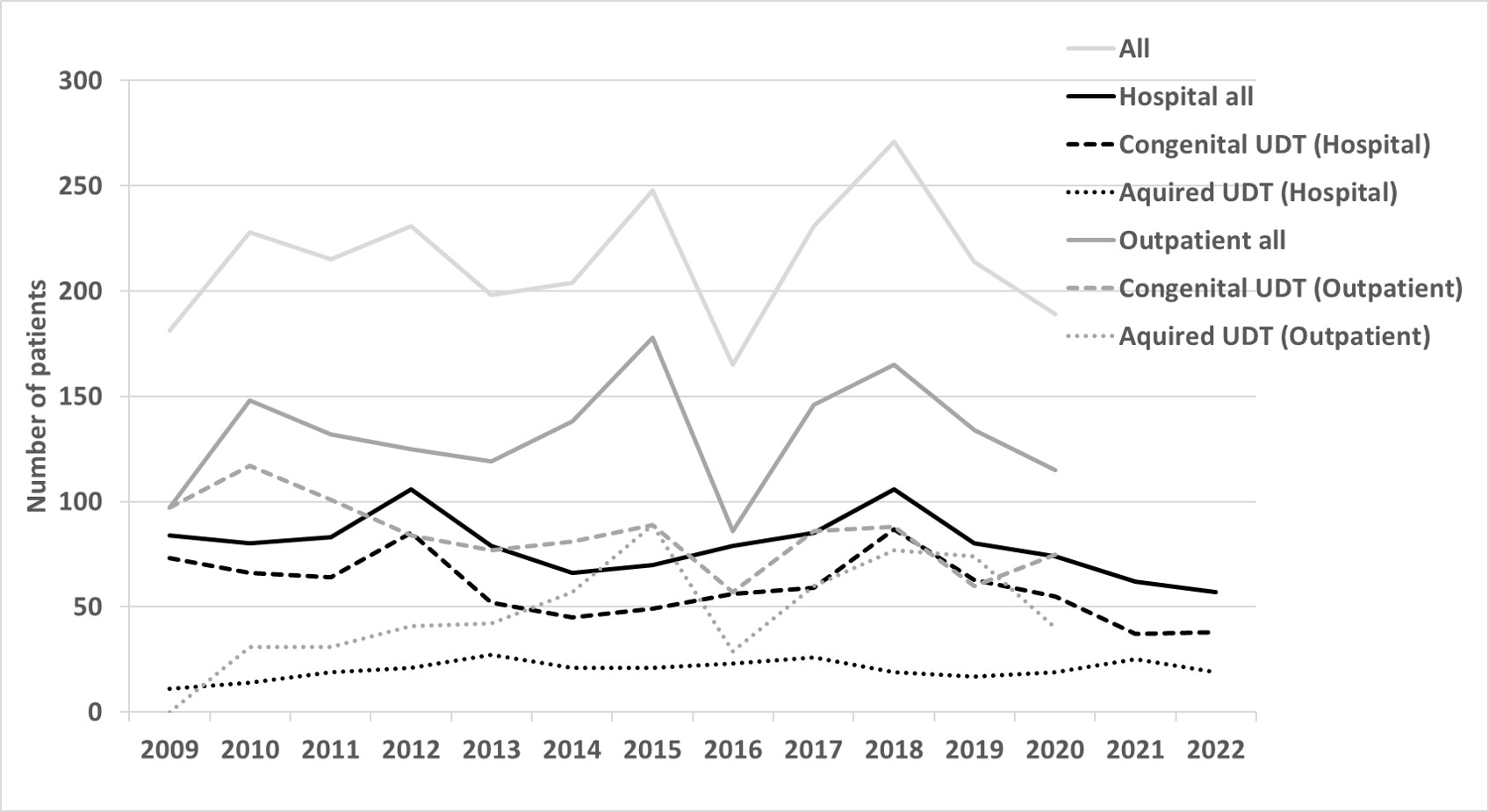

Supplement: Supplementary file 1 — Supplementary file1 (JPG 144 KB) [file 383_2024_5659_MOESM1_ESM.jpg]
