# Supplementary material for: A closer look to the timing of orchidopexy in undescended testes and adherence to the AWMF-guideline
Source: Pediatr Surg Int. 2024 Feb 29;40(1):60. doi: 10.1007/s00383-024-05659-3 (PMC10904547; doi:10.1007/s00383-024-05659-3)
Supplement: Supplementary file 2 — Supplementary file2 (DOCX 15 KB) [file 383_2024_5659_MOESM2_ESM.docx]

|  | **All years** | **2009** | **2010** | **2011** | **2012** | **2013** | **2014** | **2015** | **2016** | **2017** | **2018** | **2019** | **2020** | **2021** | **2022** |
| --- | --- | --- | --- | --- | --- | --- | --- | --- | --- | --- | --- | --- | --- | --- | --- |
| **UDT, all patients** | 1757 | 84 | 80 | 83 | 106 | 79 | 66 | 70 | 165 | 231 | 271 | 214 | 202 | 84 | 80 |
| **Median age surg [months],**  ***range*** | 27,  *7-217* | 21.5,  *7-147* | 19,  *7-191* | 32,  *7-202* | 17,  *7-165* | 15,  *7-128* | 21.5,  *7-141* | 24,  *8-167* | 29,  *7-200* | 29,  *7-196* | 30,  *7-217* | 43,  *7-174* | 28,  *7-194* | 21.5,  *7-147* | 19,  *7-191* |
| **UDT, hospital** | 1111 | 84 | 80 | 83 | 106 | 79 | 66 | 70 | 79 | 85 | 106 | 80 | 74 | 62 | 57 |
| **Median age surg [months],**  ***range*** | 23,  *7-217* | 21.5,  *7-147* | 19,  *7-191* | 32,  *7-202* | 17,  *7-165* | 15,  *7-128* | 21.5,  *7-141* | 24,  *8-167* | 23,  *7-200* | 26,  *7-196* | 29.5,  *7-217* | 30,  *8-128* | 27.5,  *7-194* | 16.5,  *7-165* | 20,  *7-169* |
| **UDT, outpts** | 646 | 97 | 148 | 132 | 125 | 119 | 138 | 178 | 86 | 146 | 165 | 134 | 115 |  |  |
| **Median age surg [months],**  ***range*** | 37,  *7-174* |  |  |  |  |  |  |  | 38,  *10-136* | 31.5,  *9-151* | 31,  *9-146* | 50.5,  *7-174* | 28,  *9-160* |  |  |
| **Congenital UDT** | 1398 | 73 | 66 | 64 | 85 | 52 | 45 | 49 | 113 | 145 | 175 | 123 | 130 | 37 | 38 |
| **Median age surg [months],**  ***range*** | 16,  *7-202* | 21,  *7-147* | 13,  *7-159* | 20,  *7-202* | 15,  *7-165* | 11,  *7-103* | 16,  *7-141* | 18,  *8-167* | 23,  *7-200* | 15,  *7-196* | 17,  *7-182* | 18,  *8-128* | 14,  *7-171* | 12,  *7-73* | 16,  *7-159* |
| **Median time refer - surg [days], *range*** | 46,  1-1836 | 32.5  1-302 | 35  *4-186* | 40,  *2-331* | 42,  *3-450* | 36,  *4-240* | 44,  *2-272* | 38,  *4-300* | 36,  *10-328* | 47,  *5-575* | 52,  *1-533* | 52,  *2-1836* | 78,  *2-1044* | 61,  *4-430* | 124.5,  *8-686* |
| **Congenital UDT, Hosp** | 829 | 73 | 66 | 64 | 85 | 52 | 45 | 49 | 56 | 59 | 87 | 63 | 55 | 37 | 38 |
| **Median age surg [months],**  ***range*** | 16,  *7-202* | 21,  *7-147* | 13,  *7-159* | 20,  *7-202* | 15,  *7-165* | 11,  *7-103* | 16,  *7-141* | 18,  *8-167* | 14,  *7-200* | 13,  *7-196* | 22,  *7-182* | 19,  *8-128* | 18,  *7-178* | 12,  *7-73* | 16,  *7-159* |
| **Median time refer - surg [days], *range*** | 42,  1- | 32.5  1-302 | 35  *4-186* | 40,  *2-331* | 42,  *3-450* | 36,  *4-240* | 44,  *2-272* | 38,  *4-300* | 36,  *10-328* | 42,  *3-575* | 42,  *1-533* | 49,  *2-248* | 71,  *4-557* | 61,  *4-430* | 124.5,  *8-686* |
| **Congenital UDT, Outpts** | 366 | 97 | 117 | 101 | 84 | 77 | 81 | 89 | 57 | 86 | 88 | 60 | 75 |  |  |
| **Median age surg [months],**  ***range*** | 16,  *8-139* |  |  |  |  |  |  |  | 27,  *10-126* | 16,  *9-124* | 13,  *9-129* | 15.5,  *8-121* | 15,  *8-121* |  |  |
| **Median time refer - surg [days], *range*** | 118,  *2-1836* |  |  |  |  |  |  |  |  | 101,  *21-304* | 118,  *5-496* | 119, *37-1836* | 158,  *2-1044* |  |  |
| **Acquired UDT** | 611 | 11 | 14 | 19 | 21 | 27 | 21 | 21 | 52 | 86 | 96 | 91 | 59 | 25 | 19 |
| **Median age surg [months],**  ***range*** | 63,  *7-217* | 27,  *12-147* | 58.5,  *15-191* | 64,  *13-134* | 65,  *9-151* | 41,  *7-128* | 45,  *14-118* | 50,  *9-148* | 68,  *13-153* | 63,  *13-182* | 72.5,  *13-217* | 71,  *7-174* | 65,  *16-194* | 53,  *17-159* | 66,  *10-169* |
| **Median time refer - surg [days], *range*** | 47,  *1-3215* | 35,  *14-1162* | 30,  *1-58* | 32,  *1-164* | 34,  *2-254* | 43,  *6-213* | 43,  *7-116* | 33,  *2-645* | 46,  *6-3215* | 55,  *3-3093* | 46,  *2-2981* | 46,  *2-2311* | 102,  *8-2564* | 83,  *3-332* | 173,  *10-483* |
| **Acquired UDT, Hosp** | 282 | 11 | 14 | 19 | 21 | 27 | 21 | 21 | 23 | 26 | 19 | 17 | 19 | 25 | 19 |
| **Median age surg [months],**  ***range*** | 55.5,  *7-217* | 27,  *12-147* | 58.5,  *15-191* | 64,  *13-134* | 65,  *9-151* | 41,  *7-128* | 45,  *14-118* | 50,  *9-148* | 64,  *17-153* | 60,  *14-182* | 70,  *13-217* | 43,  *16-117* | 49,  *16-194* | 53,  *17-159* | 66,  *10-169* |
| **Acquired UDT, Outpts** | 280 | 0 | 31 | 31 | 41 | 42 | 57 | 89 | 29 | 60 | 77 | 74 | 40 |  |  |
| **Median age surg [months],**  ***range*** | 71.5  *7-174* |  |  |  |  |  |  |  | 79,  *13-136* | 63,  *13-151* | 73,  *14-146* | 79,  *7-174* | 68.5,  *19-160* |  |  |
