# Supplementary material for: A closer look to the timing of orchidopexy in undescended testes and adherence to the AWMF-guideline
Source: Pediatr Surg Int. 2024 Feb 29;40(1):60. doi: 10.1007/s00383-024-05659-3 (PMC10904547; doi:10.1007/s00383-024-05659-3)
Supplement: Supplementary file 3 — Supplementary file3 (DOCX 12 KB) [file 383_2024_5659_MOESM3_ESM.docx]

|  | Congenital UDT n=882 (%) | Acquired UDT n=247 |
| --- | --- | --- |
|  |  |  |
| Epifascial | 262 (29.6%) | 89 (36.0%) |
| Inguinal low | 131 (14.9%) | 61 (24.7%) |
| Inguinal Middle | 255 (28.9%) | 75 (30.4%) |
| Inguinal high | 110 (12.5%) | 22 (8.9%) |
| Abdominal | 124 (14.1%) | 0 (0%) |
